# Sleep Deprivation Is Associated With Increased Risk for Hypertensive Heart Disease: A Nationwide Population-Based Cohort Study

**DOI:** 10.7759/cureus.33005

**Published:** 2022-12-27

**Authors:** Endurance O Evbayekha, Henry O Aiwuyo, Arthur Dilibe, Bede N Nriagu, Abiodun B Idowu, Ruth Y Eletta, Evidence E Ohikhuai

**Affiliations:** 1 Internal Medicine, St. Luke's Hospital, Chesterfield, USA; 2 Internal Medicine, Brookdale University Hospital Medical Center, Brooklyn, USA; 3 Internal Medicine, East Carolina University (ECU) Health, Greenville, USA; 4 Internal Medicine, New York Medical College, Metropolitan Hospital Center, New York City, USA; 5 Internal Medicine, Einstein Medical Center Philadelphia, Philadelphia, USA; 6 Pediatrics, Woodhull Medical Center, Brooklyn, USA; 7 Public Health, Jackson State University, School of Public Health, Jackson, USA

**Keywords:** sleep hygiene, sleep debt, deprivation, hypertensive heart disease, sleep, sleep deprivation

## Abstract

Background: Literature documenting the in-hospital cardiovascular outcomes of sleep deprivation (SD) patients is scarce. We aimed to compare inpatient cardiovascular outcomes in patients with sleep deprivation and those without sleep deprivation.

Method: We queried the National Inpatient Sample (NIS) database from 2016 to 2019 to conduct a retrospective observational study. Using the International Classification of Diseases, Tenth Revision (ICD-10) codes, we identified patients with sleep deprivation (SD) diagnosis and compared them to their counterparts without sleep deprivation (NSD). The cardiovascular outcomes of interest were hypertensive heart disease (HHD), atrial fibrillation (AF), and ST-segment and non-ST-segment elevation myocardial infarction (STEMI and NSTEMI, respectively). We used multivariable regression analysis to unearth the relationship between sleep deprivation and cardiovascular disease.

Results: There were 28,484,087 patients admitted during the study period, among which 2.1% (6,08,059) with a mean age of 59 (sd=19) years had a sleep deprivation diagnosis unrelated to medical or psychiatric illness. Of these, 75.7% were Caucasians, 11.5% were Blacks, and 8% were Hispanics. Individuals with sleep deprivation had a higher odds ratio (OR) of HHD, i.e., OR=1.3 (1.29-1.31), p<0.0001. The odds of heart failure with reduced ejection fraction (HFrEF) was 0.9 (0.9-1.92), p=0.45; heart failure with preserved ejection fraction (HFpEF) was 0.98 (0.97-1.01), p=0.31; and the odds of the SD population for AF was 0.9 (0.89-1.03), p=0.11.

Conclusion: Sleep deprivation seems to be more prevalent in the Caucasian population. Individuals with sleep deprivation have a higher risk of hypertensive heart disease but similar outcomes to the general population in terms of AF, HFrEF, and HFpEF.

## Introduction

Sleep is an integral part of an individual’s physical and psychological well-being. Insufficient sleep has been linked to numerous adverse health outcomes, ranging from mood disorders, reduced performance, cognitive impairment, increased propensity for accidents, and elevated cardiovascular risk [1]. However, there seems to be limited data exploring the deleterious effects of sleep deprivation in hospitalized patients. The National Academy of Medicine estimates that hundreds of billions of dollars per year are spent caring for patients with sleep disorders, and it is estimated that nearly 50-70 million Americans suffer from a sleep disorder [1]. This significant disease burden on the individual and the healthcare system at large highlights the importance of our focus on this area of study.

Definition and classification

Sleep deprivation or sleep insufficiency is defined as a state when sleep is insufficient to support adequate alertness, performance, and health, either because of reduced total "sleep time" (quantity) or an interruption in sleep by frequent arousals (quality) [2]. Sleep insufficiency can be classified as either acute or chronic, with the former being a reduction in total sleep time within a 48 hours period and the latter being a state of a long-standing sub-optimal amount of sleep required for healthy well-being. It is pertinent to note that chronic sleep deprivation is distinct from insomnia, given that, unlike insomnia, sleep-deprived individuals will fall asleep if given the opportunity [2]. While most studies generally recommend six to eight hours of nighttime sleep, there exist significant interindividual differences, with some individuals thriving on less and others requiring more.

Disease burden (epidemiology)

It is paradoxical that patients hospitalized with acute illness who arguably are in need of adequate and quality sleep for restorative function suffer consequential poor sleep while in the hospital. There exist some peculiarities in sleep deprivation seen in hospitalized patients compared to that seen in the general population. Some of the factors that contribute to the impairment of sleep quality in inpatient settings include pre-hospital diagnoses like obstructive sleep apnea (OSA); adverse effects of medications; frequent arousals triggered by noise from hospital staff, overhead pagers, or alarms from medical equipment; patient care interventions like vital signs or blood draws; interruptions by housekeeping staff for routine linen management, waste management, and infection control; and poor sleep quality related to pain and unfamiliar environment. Although there exist limited data on the burden of sleep deprivation and its deleterious effects on hospitalized patients, multiple studies show a high prevalence of sleep deprivation in these subsets of patients. In the study by Frighetto et al., the prevalence was estimated to be as high as 50% [3]. Similarly, an observational study by Manian et al. reported a ≥50% rate of sleep deficiencies in 1,238 hospitalized patients with infectious diseases [4].

Pathophysiology

While we shed a general light on the gruesome problem of sleep deprivation in hospitalized patients, our study seeks to specifically explore the adverse cardiovascular outcomes in these patients that either develop or worsen due to poor sleep. Epidemiological research has long established a link between sleep deprivation and cardiometabolic risk. Multiple mechanisms involving physiology, genetics, and behavior have been proposed. Still, one hypothesis that has been well accepted is that sleep deprivation is associated with a proinflammatory state. Experimental studies have shown that an acute decrease in the duration of sleep by a few hours over the course of several days causes an increase in proinflammatory markers like c-reactive protein (CRP), which is believed to play a role in the pathogenesis of metabolic and cardiovascular diseases via inflammatory activation and vascular endothelial dysfunction. Other proposed mechanisms include impairment of the hypothalamic-pituitary-adrenal axis and dysregulation of the autonomic nervous system [2].

With sleep deprivation, there is also a disturbance in the normal circadian pattern of blood pressure. Blood pressure normally fluctuates over a 24-hour period, dips at night during rest/sleep, and undergoes a steep rise in the morning, eventually peaking in the afternoon. In hospitalized patients with sleep deprivation, there is a lack of that nocturnal dip in systolic and diastolic blood pressure, and we see a persistently activated sympathetic nervous system that results in elevated blood pressure [5]. This disruption in the normal sleep-wake-cycle can increase the risk of newly developed hypertension or worsening pre-existing elevated blood pressures.

Cardiovascular diseases (CVD) are a broad group of tightly linked disorders that cause health problems and death. Broadly speaking, CVDs incorporate a long list of diseases involving the heart or blood vessels. Our study generally focuses on the interplay between poor sleep in hospitalized patients and a subset of outcomes like hypertension, atrial fibrillation, coronary artery disease, and heart failure. As previously highlighted, the pathway to outcomes like hypertension is one of the "direct" pathophysiological deleterious effects of sleep deficiency that can be "easily" explained. However, there might be other less explored "indirect" ways through which sleep deprivation in an inpatient setting can increase cardiovascular morbidity and mortality. We believe that more studies need to be done to evaluate the impact of poor sleep in the studied demography and how decreased patient satisfaction, increased length of hospital stay, mood, and circadian dysfunction affect patient outcome, if at all, in the post-hospital discharge phase.

Manifestations

The signs and symptoms of sleep deprivation observable in hospitalized patients can be multivariate in nature. The earliest (and probably easiest) to recognize is excessive daytime sleepiness in these patients. Evidence in the field of sleep study suggests that only the last two stages of sleep (deep sleep and rapid eye movement {REM} sleep) are considered restorative. When patients experience frequent interruptions in their sleep, they spend less time during “the restorative phase” of sleep, which leads to a vicious cycle of prolonged sleep duration that does not lead to a “well-rested state.” Such patients experience fatigue, irritability, excessive daytime sleepiness, decreased desire to participate in recovery activities, and ultimately an increased length of hospital stay. Other manifestations of sleep deprivation in hospitalized patients include delirium, increased pain perception, increased blood sugars, and increased blood pressure [5,6].

Diagnosis, treatment, and prognosis

Sleep deprivation-related cardiovascular outcomes management generally revolves around interventions that improve sleep during the hospital stay. Unfortunately, there is no ultimate elixir that completely addresses the problem of sleep deprivation in hospitalized patients. We believe that increased awareness of the problem is a good starting point. In hospitalized patients, it is prudent to consider the distinction between sleep deprivation caused by diagnosed and undiagnosed sleep disorders, e.g., obstructive sleep apnea (OSA) versus sleep deprivation caused by frequent sleep interruptions or patient care interventions. For the latter, a variety of non-pharmacologic interventions (noise reduction, light therapy, and reducing nighttime interruptions) versus pharmacological sleep aids (e.g., melatonin) can be helpful. While patients with obstructive sleep apnea will be better managed with positive airway pressure (PAP) therapy. The overall approach will be tailored to the specific needs of the patients [2].

Lastly, rather than merely addressing the problems as they arise, as clinicians and healthcare workers, we believe that the best approach is preventative rather than curative. While admitting that this is undoubtedly a daunting task, there are easy steps we can all incorporate into our daily practice, like avoiding medications that distort the sleep architecture, avoiding non-emergent interventions at night that can wait till morning, and only scheduling "AM labs" if truly appropriate and necessary [7,8].

## Materials and methods

We retrospectively analyzed the National Inpatient Sample (NIS) database using the hospital discharge data provided by the Healthcare Cost and Utilization Project - National Inpatient Sample. We identified all persons admitted to the inpatient setting with a primary or secondary diagnosis of sleep deprivation. We compared the incidence of cardiovascular outcomes with their counterparts who did not have sleep deprivation. Our study time frame focused on the dataset from 2016 to 2019 because the International Classification of Diseases, Tenth Revision (ICD-10) codes became active from 2015 onwards. Furthermore, the 2016 to 2019 dataset is the most recent dataset available, and no trend weight is needed to create a national estimate for the NIS dataset from 2012 and beyond. The National Inpatient Sample is publicly available. It is a deidentified database that correlates to about eight million hospitalizations annually, thus about 20% of all hospitalizations across the United States, representing 20% of all admissions across the United States. The NIS dataset represents individual hospitalizations and holds clinically useful data, including diagnosis and past and present medical and surgical histories. Patients 18 years or older with a primary or secondary diagnosis of "sleep deprivation or insomnia" were included in the study using the International Classification of Diseases, Tenth Revision-Clinical Modification (ICD-10-CM) diagnosis code (The Healthcare Cost and Utilization Project {HCUPS}) [9].

Study outcomes

The cardiovascular outcomes of choice were hypertensive heart disease (HHD), atrial fibrillation (AF), ST-segment and non-ST-segment elevation myocardial infarction (STEMI and NSTEMI, respectively), heart failure with preserved and reduced ejection fraction (HEpEF and HErEF, respectively), stroke, transient ischemic attack (TIA), and pulmonary embolism (PE).

Inclusion and exclusion criteria

The study included patients aged 18 years or older admitted with a primary or secondary sleep deprivation diagnosis. The ICD-10 codes have been previously validated by Mader et al. [10]. We excluded sleep deprivation due to medical (including sleep apnea) or psychiatric conditions. We excluded all patients younger than 18 years and those transferred from the hospital to an outside hospital for any reason.

Analysis

Data from 2016 to 2019 were used for statistical analyses. We preferred the 2016 and beyond datasets because they are the most recent dataset and used the ICD-10 codes in contrast to datasets before 2015. Also, the 2016-2019 dataset was redesigned to overcome discrepancies that plague the dataset of prior years, such as the exclusion of long-term care facilities and sample stratification by nine census divisions rather than four. The 2016-2019 NIS database was pooled from 48 states, including Maryland, representing 97% of the United States population, making the NIS the largest inpatient database [9]. We applied a multivariable regression model, a descriptive statistical method for demographics and baseline characteristics of patients, which are presented as percentages. We reported adjusted odds ratio (OR) and confidence intervals (CI). 

Variables and significance

We included demographic and socioeconomic factors, such as age, sex, ethnicity, median household income, hyperlipidemia, obesity, type 2 diabetes, and smoking. To overcome confounders, we applied propensity matching and controlled for the above-mentioned variables. We also controlled for insomnia disorders and sleep apnea. Results with p-values of <0.05 were adopted as statistically significant. All analyses were performed using Statistical Analysis System (SAS) software version 9.4 (SAS Institute Inc., Cary, NC).

## Results

**Figure 1:**
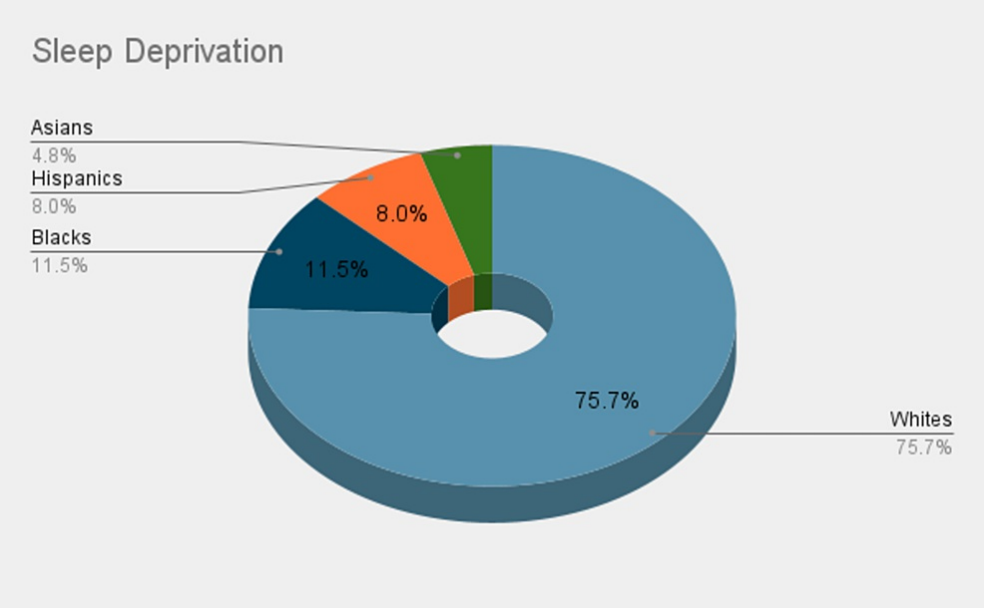
In-hospital distribution of sleep deprivation according to ethnicity.

There were 28,484,087 subjects over our study period, of which 2.1% (6,08,059) were diagnosed with sleep deprivation, with a male predominance of 56% and a mean age of 59 (sd=19) years. According to prevalence by ethnicity, 75.7% were Whites, 11.5% were Blacks, and 8% were Hispanics (Figure 1).

Among the population with sleep deprivation, there were 38,913 (6.4%) individuals with heart failure and preserved ejection fraction (HFpEF) 0.98 (0.97-1.01), 36,328 (5.9%) with heart failure and reduced ejection fraction (HFrEF) 0.9 (0.9-1.92), 2,448 (0.4%) with ST-segment elevated myocardial infarction (STEMI) 0.52 (0.5-1.60), 10,426 (1.7%) with non-ST-segment elevation myocardial infarction (NSTEMI) 0.7 (0.68-1.71), 2,691 (0.4%) had a transient ischemic attack (TIA) 0.8 (0.7-1.91), 84,111 (13.8%) with atrial fibrillation (AF) 0.9 (0.89-1.03), 7,196 (1.2%) with pulmonary embolism (PE) 1.04 (0.9-1.04), and 8,871 (1.4%) had a stroke 0.7 (0.6-1.7). All the previously listed comorbidities had no statistical significance during the analysis, i.e., p>0.05.

A significant proportion of the population diagnosed with sleep deprivation also had hypertensive heart disease (HHD) 40% (2,43,139). There was a statistically significant associated increase in odds for hypertensive heart disease odds ratio 1.3 (1.29-1.31), p<0.0001. Table 1 summarizes the findings from our analysis.

**Table 1:** The comorbidity burden of individuals with sleep deprivation. HFpEF: heart failure with preserved ejection fraction; HFrEF: heart failure with reduced ejection fraction; HHD: hypertensive heart disease; STEMI: ST-segment elevation; NSTEMI: non-ST-segment elevation; TIA: transient ischemic attack; AFIB: atrial fibrillation; PE: pulmonary embolism; CI: confidence interval The total number of individuals with sleep deprivation diagnoses was 6,08,059.

Comorbidity	Number of Individuals with comorbidity	Percentage	Odds ratio (CI)	p-Value
HFpEF	38,913	6.4%	0.98 (0.97-1.01)	0.31
HFrEF	36,328	5.9%	0.9 (0.90-1.92)	0.45
HHD	2,43,139	39.9%	1.3 (1.29-1.31)	0.0001
STEMI	2,448	0.4%	0.52 (0.50-1.60)	0.12
NSTEMI	10,426	1.7%	0.70 (0.68-1.71)	0.86
TIA	2,691	0.4%	0.80 (0.70-1.91)	0.23
AFIB	84,111	13.8%	0.90 (0.89-1.03)	0.11
PE	7,196	1.2%	1.04 (0.90-1.04)	0.70
Stroke	8,871	1.4%	0.70 (0.60-1.70)	0.50

## Discussion

Sleep is a key component of human physiology that causes transient inactivity of the central nervous system presenting as suspension of consciousness [11]. It occurs naturally in humans and animals with age-related differences in sleep duration [12]. Sleep is a highly regulated process and hence subject to hormonal regulation. The evidence supporting the adverse impact of sleep deprivation on the cardiovascular system is robust [13-16]. This study focuses on the effects of sleep deprivation and its impact on specific cardiovascular outcomes.

Hypertension is a significant traditional cardiovascular risk factor that causes geometric changes in myocardial tissues and increased wall stress on the heart [17]. Several studies have shown relaxation techniques to improve the quality of blood pressure control [18]. Sleep causes a significant reduction in muscle activity and reduces overall myocardial workload. This effect is expected to reduce strain on the heart and vasculature. There is evidence to support accelerated atherosclerosis among patients who are sleep-deprived, and this increases the risk for fatal and non-fatal cardiovascular (CV) outcomes [18].

Hypertensive heart disease is a clinical spectrum of disorders ranging from concentric remodeling, concentric hypertrophy, and eccentric hypertrophy of the left ventricle [17]. Some studies have shown a significant association between sleep deprivation, poor blood pressure control, and the development of end-organ damage [19,20]. Among in-patient subjects selected for this study, we observed that the odds of hypertensive heart disease were higher in sleep-deprived patients than in non-sleep-deprived patients. This was identified among patients who either had a primary or secondary diagnosis of sleep deprivation at the time of discharge. This finding reflects the possibility that chronically sleep-deprived individuals are more likely to have uncontrolled blood pressure than non-sleep-deprived individuals.

Researchers have observed an increased cortisol level among patients with sleep deprivation, which has been noted responsible for some degree of fluid retention and worsening extracellular fluid volume, leading to uncontrolled hypertension [21]. Chronically, it can increase afterload, leading to remodeling of the left ventricular structure and the development of hypertensive heart disease.

There is evidence that levels of vasoconstrictors like endothelin are increased in sleep-deprived individuals, which can cause elevated blood pressure [22]. Other studies on sleep-deprived patients have established elevated counterregulatory hormones among patients who have shortened or absent rapid eye movement (REM) sleep [23]. This aspect of sleep regulates central nervous system activities during sleep. If it is reduced for any reason, it increases norepinephrine levels and predisposes to adverse cardiovascular events. Pro-inflammatory cytokines like IL 1, 6, and tumor necrosis factor (TNF) alpha are elevated in sleep-deprived individuals. This contributes significantly to the inflammatory component of vascular diseases, including strokes, and coronary artery disease. 

Major cardiovascular events are usually encountered in the mornings because of increased platelet vasoreactivity and elevated levels of counter-regulatory hormones, which causes vasoconstriction [24,25]. Sleep-deprived patients who already have increased levels of these hormones are more likely to develop negative CV outcomes than patients who are not sleep-deprived.

Neuronal nitric oxide synthase is severely reduced in sleep-deprived patients; this knocks out vasodilatory benefits and elevates nocturnal blood pressure, predisposing to hypertensive heart disease. It has been observed that people with shorter sleep duration have lower levels of gamma amino butyric acid (GABA) which is a major inhibitory neurotransmitter produced by the limbic system [24-26]. This inadvertently leads to a decreased central inhibition of motor activities of the body, causing increased tone and demand on the heart during sleep.

Our study also showed no significant difference between both cohorts of patients regarding the risk of atrial fibrillation. Hypertensive heart disease is associated with various electrical abnormalities of the heart. The presence of increased levels of cardiostimulatory hormones like catecholamines in patients with poor sleep can further increase the propensity for developing new-onset arrhythmias or worsening arrhythmia burden for patients already predisposed. Some studies have shown a higher prevalence of atrial fibrillation among patients with sleep apnea [27-29]. The use of telemetry to establish the burden of arrhythmia in sleep-deprived patients is, therefore, plausible.

Our study did not show any difference in the prevalence of HFrEF, stroke, or acute coronary syndromes among sleep-deprived versus non-sleep-deprived. Some studies have found increased rates of major adverse cardiovascular outcomes among patients with sleep deprivation [16,30]. Our patient population, centered on in-patients, may have been responsible for the lack of statistical difference compared with the general population. To clarify this argument further, there is a need to obtain large prospective population studies.

Strength of the study

In this study, we discussed the implications of sleep deprivation with regard to cardiovascular health. Since this was a retrospective analysis of records, there is less likelihood of recall or confirmation bias. The NIS is the largest database for inpatient records. It provides a great sample size and statistical power. Lastly, research on the cardiovascular outcomes in this population has not been well established; hence our study will serve as a great addition to the current body of knowledge.

Limitations of the study

The NIS database is useful for research purposes. However, it is a core administrative tool for billing and hence the possibility of overbilling, underbilling, and wrong coding. There are also some missing frequencies during some analyses that may impact analysis (albeit less likely). The NIS cannot differentiate between the inter-hospital transfer of patients during the same hospitalization course and hence can result in duplication.

## Conclusions

Our findings emphasize the need for healthcare professionals to be advocates for promoting quality sleep experiences for their patients, as there is evidence to support an association between poor sleep and the tendency to develop hypertensive heart disease. Overall, sleep neglect may be as bad as cardiovascular neglect, and factors that improve the quality and quantity of sleep should be addressed among patients with hypertension. This data must be interpreted cautiously as sleep deprivation is likely higher in the outpatient diagnosis and setting. Hence, more studies are warranted to establish the burden of cardiovascular disease in individuals with chronic sleep deprivation.
